# Health sciences librarians' engagement in open science: a scoping review

**DOI:** 10.5195/jmla.2021.1256

**Published:** 2021-10-01

**Authors:** Dean Giustini, Kevin B. Read, Ariel Deardorff, Lisa Federer, Melissa L. Rethlefsen

**Affiliations:** 1 dean.giustini@ubc.ca, University of British Columbia, Biomedical Branch Library, Vancouver General Hospital, Vancouver, British Columbia, Canada; 2 kevin.read@usask.ca, University of Saskatchewan, Health Sciences Library, Saskatoon, Saskatchewan, Canada; 3 ariel.deardorff@ucsf.edu, University of California, San Francisco Library, San Francisco, CA; 4 lisa.federer@nih.gov, NLM Data Science and Open Science Librarian, National Library of Medicine, Bethesda, Maryland; 5 mlrethlefsen@gmail.com, University of New Mexico, Health Sciences Library & Informatics Center, Albuquerque, NM

**Keywords:** health sciences libraries, health sciences librarians, open science

## Abstract

**Objectives:**

To identify the engagement of health sciences librarians (HSLs) in open science (OS) through the delivery of library services, support, and programs for researchers.

**Methods:**

We performed a scoping review guided by Arksey and O'Malley's framework and Joanna Briggs' Manual for Scoping Reviews. Our search methods consisted of searching five bibliographic databases (MEDLINE, Embase, CINAHL, LISTA, and Web of Science Core Collection), reference harvesting, and targeted website and journal searching. To determine study eligibility, we applied predetermined inclusion and exclusion criteria and reached consensus when there was disagreement. We extracted data in duplicate and performed qualitative analysis to map key themes.

**Results:**

We included fifty-four studies. Research methods included descriptive or narrative approaches (76%); surveys, questionnaires, and interviews (15%); or mixed methods (9%). We labeled studies with one or more of FOSTER's six OS themes: open access (54%), open data (43%), open science (24%), open education (6%), open source (6%), and citizen science (6%). Key drivers in OS were scientific integrity and transparency, openness as a guiding principle in research, and funder mandates making research publicly accessible.

**Conclusions:**

HSLs play key roles in advancing OS worldwide. Formal studies are needed to assess the impact of HSLs' engagement in OS. HSLs should promote adoption of OS within their research communities and develop strategic plans aligned with institutional partners. HSLs can promote OS by adopting more rigorous and transparent research practices of their own. Future research should examine HSLs' engagement in OS through social justice and equity perspectives.

## INTRODUCTION

Open science (OS) is a global movement that promotes transparency and reproducibility in research, contributes to biomedical education and training, and facilitates secondary research [1]. In recent years, health sciences librarians (HSLs) have participated in a range of OS projects, but to date there have been no broader studies identifying this complex and varied support. To address this gap, we conducted a scoping review of HSLs' efforts to support OS researchers and advance OS principles through library-led services and programs.

### Definitions and background

The approach, methodology, and analysis of this review was informed by several definitions. OS has been defined as “the practice of making everything in the discovery process fully and openly available, creating transparency, and driving … discovery by allowing others to build on existing work. The six … pillars of open science are: open data, open access, open methodology, open source, open peer review, and open education” [2]. OS also refers to an array of practices promoting openness, integrity, and reproducibility in research [3]. A glossary of terms used in this paper is available on the Open Science Framework (OSF) [4].

Vicente-Saez and others embarked on a systematic review to develop a better definition of OS [5]. After reviewing thirty-six studies, the authors defined OS as “transparent and accessible knowledge that is shared and developed through collaborative networks” [5]. Several organizations are also working to describe and classify OS concepts and activities. To map the scope of OS activities, FOSTER (Facilitate Open Science Training for European Research), a European Union (EU)–funded project, developed a taxonomy that divides OS into principles and practices [6]. OS principles, including increased transparency, reuse, participation, cooperation, accountability, and reproducibility in research, aim to improve the quality and reliability of research through principles like inclusion, fairness, equity, and sharing [7]. Open practices refer to the way science is conducted, such as open access to research publications, data sharing, open notebooks, transparency in research evaluation, open peer review, ensuring reproducibility of research (where possible), transparency in methods, open source code, software and infrastructure, citizen science, and open educational resources [7].

Definitions and views outside of North America and Europe are also important to our understanding of OS. Especially useful are perspectives from Africa and Latin America. As Sarcina says, OS “may take different shades according to geographic perspectives across nations and regions [and] can differ according to the stakeholders and actors involved” [8]. One paper involving Rwandan librarians makes a plea to include African voices in OS and to guard against excessive input from countries where OS originates and is well resourced [9].

### Building library support and rationale

HSLs have played key roles in supporting open scholarship [10, 11]. As partners in promoting OS, HSLs have increased their participation in research data management [12], open access (OA) initiatives [13–15], and citizen science [16]. Since 2010, the EU and member states have implemented open data policies [17], and librarians have increased their engagement by improving OS governance and e-infrastructure [18]. For HSLs serving biomedical researchers, promoting OA and open data practices is key to achieving an open, inclusive, and equitable science ecosystem [19]. The Medical Library Center of New York was an early advocate of open scholarship and “the FAIR guiding principles for scientific data management and stewardship” (making data findable, accessible, interoperable, reusable) [20].

Our objectives in this review are to assess the scope of HSLs' support of OS, examine strategic approaches they have taken, and identify the impact of library services in OS. We are interested in how HSLs develop OS services, demonstrate their impact over time, and align user services with broader institutional goals and resources via partnerships, curriculum integration, policies, and infrastructure.

## METHODS

Our scoping review was guided by Arksey and O'Malley's framework [21] and Joanna Briggs' Manual for Scoping Reviews [22]. We developed a review protocol to guide the process and shared it via the OSF in March 2020 [4]. For reporting, we used PRISMA-S for our searches [23] and PRISMA-ScR for the review itself [24].

The following research questions (RQ) guided our review:

- **RQ1: Actions, barriers, and drivers**: What drivers affect HSLs' participation in OS? How do HSLs integrate OS service models into their broader institutional missions and strategic initiatives?
- **RQ2: Services and support:** What types of OS services and support do HSLs provide for researchers and other users within their institutions? How are HSLs' OS services evaluated?
- **RQ3: Roles and stakeholders:** What roles do HSLs play in support of OS in their library settings and institutions? Who are the key stakeholders that HSLs collaborate and partner with when providing OS services and support?

In our review, the term “researcher” refers to any scientist, clinician or practitioner, student, or other individual working in a hospital or health care setting, biomedical lab, government agency, or academic or research institution who is engaged in research or in the process of learning how to conduct research.

### Data sources and literature searching

Scoping reviews employ broad exploratory searches of relevant studies to determine key characteristics of a subject [25]. We searched five bibliographic databases from 2010 to 2020: MEDLINE (Ovid); Embase (Ovid); CINAHL (EBSCO); Library, Information Science and Technology Abstracts (LISTA) (EBSCO); and the Web of Science Core Collection (Science Citation Index Expanded, Social Sciences Citation Index, Arts and Humanities Citation Index, Conference Proceedings Citation Index-Science, Conference Proceedings Citation Index-Social Sciences and Humanities, Emerging Sources Citation Index).

We limited our searching for the ten-year period from 2010 to 2020 because OS, as an umbrella term of open practices beyond open access, was not widely discussed before 2010. Although some public access policies, such as the National Institutes of Health (NIH) Public Access Policy, were established before 2010, the first studies examining their application in HSLs began to appear in 2010. In our early scoping searches, we also found a high number of relevant studies published after 2015.

In addition to database searching, we searched seven journals in health sciences librarianship and three association websites. We performed reference harvesting and citation searching in Web of Science and Google Scholar using a file of highly cited peer-reviewed and grey literature found in our searches (Supplemental file S1). Searches were conducted in March 2020 and updated monthly until December 2020.

### Search strategy development

Our search terms were developed based on our RQs. We grouped them into three concept blocks: 1) librarians, libraries, and variations of “health sciences librarians or libraries”; 2) research trends in OS inclusive of our definitions, pillars, practices, principles, tools, and platforms [2, 3, 5–8]; 3) HSLs' actions, roles, and support and outcomes of providing library services and programs in OS (Supplemental file S2).

We devised our search sets in MEDLINE using two main concept blocks (health sciences librarians and OS) and tested the third group of concepts. We used the third concept block in LISTA only as it reduced search sensitivity too much in the other databases. We imposed no limits by language or study type but limited to studies published from 2010 to 2020.

Searches were performed by two authors (DG, KR) and peer reviewed by two other authors (LF, MR) using the Peer Review of Electronic Search Strategies checklist [26]. We created strategies in MEDLINE and Embase on Ovid and adapted them to the other databases and platforms. We include our MEDLINE search strategy (Supplemental file S3) and complete details of the other searches via the OSF platform [4].

### Key journals, association websites, meetings, and abstract searching

We searched meeting abstracts at websites for the Canadian Health Libraries Association, the European Association for Health Information and Libraries, and the Medical Library Association (Abstracts for Annual Meetings; login required) from 2010 to 2020.

We conducted manual searches for studies by scanning the tables of contents of seven HSL journals: the *Journal of the European Association for Health Information and Libraries, Evidence Based Library and Information Practice, Journal of the Canadian Health Libraries Association, Journal of the Medical Library Association, Journal of eScience Librarianship, Medical Reference Services Quarterly*, and *Hypothesis*.

During presearching, we learned that LIBER (Ligue des Bibliothèques Européennes de Recherche—Association of European Research Libraries) published a roadmap outlining the specific actions libraries can take to promote OS [27]. We located several relevant studies and reports in LIBER's peer-reviewed journal *LIBER Quarterly: The Journal of the Association of European Research Libraries*. We also searched three widely known open repositories—Figshare, Zenodo, and OSF—because these repositories store preregistered and finalized library research.

### Environmental scanning

We performed an environmental scan of OS-related library activities, workshops, and conferences as reported on library and university web pages, subject guides, and association websites in North America, Europe, and Australia [4]. We also performed early exploratory searches for perspectives from Latin America in LILACS and SciELO and from continental Africa in the WHO Global Index Medicus. Our environmental scan helped to identify an overall search strategy as well as search terms, key concepts, and sources of evidence most relevant to the review.

### Citation management

**Figure 1:**
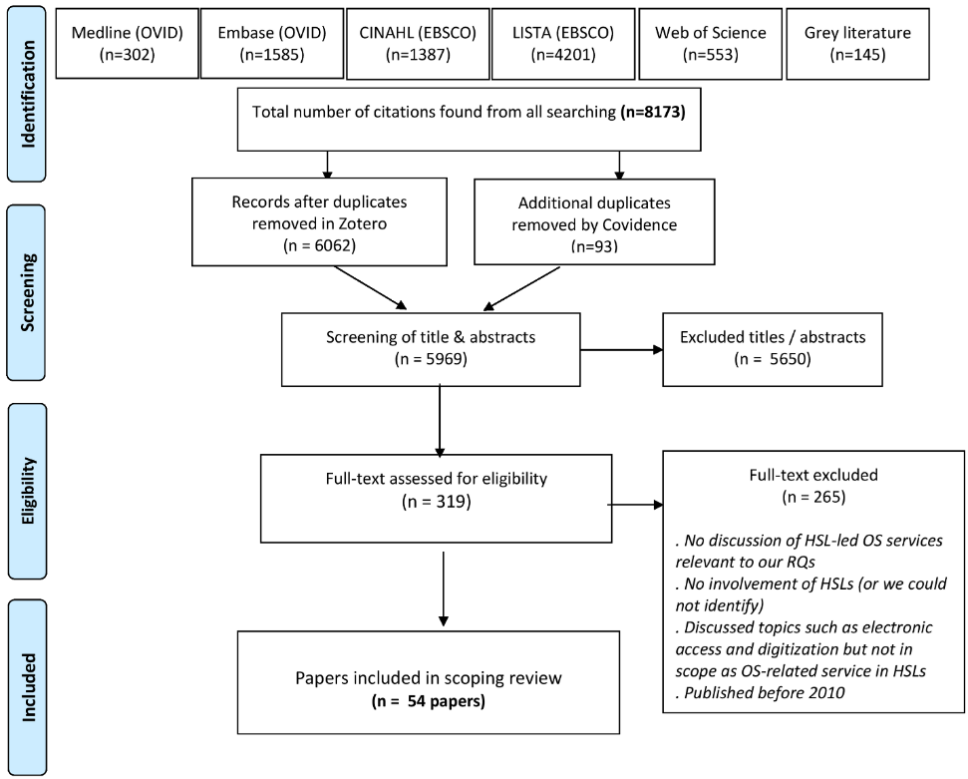
PRISMA flow diagram

We used Zotero, an open source tool, for citation management. Our searches yielded 8,173 studies from database, journal, and conference searching inclusive of reference harvesting and citation tracking [4]. The PRISMA-ScR flow diagram is shown in Figure 1. We performed deduplication in Zotero and imported unique citations into Covidence for screening.

### Our open practices as researchers

As a team of researchers, we are committed to OS principles and make our literature searches and datasets open, transparent, and publicly available. Our methodology aims to adhere to open principles as much as possible, which was increasingly important given that we conducted the study during COVID-19.? However, although we used open-source alternatives when and where possible, we relied on library subscriptions, proprietary products, and platforms to perform the bulk of the review.

### Inclusion and exclusion criteria

We considered all sources to allow for inclusion of any and all types of studies (including peer-reviewed and non-peer-reviewed studies) and methodologies (e.g., case reports, mixed methods, qualitative studies, quantitative studies). We included studies that were written in or could be translated into English.

Studies were included when they discussed the following topics:

- OS library services or open initiatives provided by HSLs on a university campus, hospital, or academic health center;
- Assessment or evaluation of OS services and support, best practices, and benchmarking; or
- HSLs' engagement and involvement in institution or campus-wide committees, working groups, institutional partnerships, infrastructure development, or policy development.

Studies and other literature were excluded if they were published before 2010 or did not discuss or describe:

- OS library services, leadership, or support in any way relevant to our research questions;
- OS-related library services aligned with our definitions of OS (e.g., discussed data management training but not for improved transparency or reproducibility);
- Digitization or accessibility initiatives as an OS-related library service; or
- Involvement of HSLs or health sciences libraries.

### Study selection and screening

We used a two-step process for screening, first considering titles and abstracts only and then moving to full-text review.

#### Title and abstract screening

After deduplication in Zotero, we moved the citations to Covidence for screening. Covidence found a further ninety-three duplicates, which were removed. We independently coded and assessed the titles and abstracts of retrieved citations in duplicate using the predetermined selection criteria. Reviewers were not blinded to author or journal names. When a title or abstract was not in English, we included it and ordered translations using one of our review member's institutional translation services. After basic screening was completed, we met to discuss references selected for inclusion. We resolved our disagreements through discussion and majority vote.

#### Full-text screening

We identified 319 studies for full-text review. We downloaded the documents into Covidence from OA journals and repositories and by accessing our institutional journal subscriptions and interlibrary loan services. For full-text screening, we adopted a process similar to that employed during title and abstract screening. Pilot testing involved assigning a random set of studies to each reviewer and then discussing and improving the process as a team. We resolved disagreements between reviewers during our regular meetings and tracked our decisions to include or exclude in Covidence.

### Data extraction

Data extraction for a scoping review is referred to as “data charting” [28]. We tested a data extraction form in Google Sheets and made changes based on this testing. Each reviewer was then assigned a second larger sample set of studies to review. We iteratively improved the form to include the following data fields: citation, author country, publication type, language, study aims, study methods, population, setting, drivers, service type, OS category, service impact, institutional integration, recommendations, and notes (Supplemental file S4). We extracted information from full-text publications and materials by taking into account each variable.

### Thematic analysis and categorization

We analyzed the studies using a method by Braun and Clarke [29]. Following the step-by-step guidelines in their method (familiarization with the data; generation of initial coding; searching, reviewing, and defining themes [29]), we identified and highlighted certain themes and patterns across the studies. To focus on identifying, analyzing, and reporting patterns within the data, the work of Thomas and Harden informed our thematic and narrative synthesis [30]. In analyzing each paper, we noted emergent themes based on study participants, types of library services and programs provided, and study outcomes. We coded the themes and placed them into categories using FOSTER's taxonomy in order to apply a consistent, structured set of terms. The taxonomy provides an overview of core topics in OS and is used by FOSTER to organize teaching and learning materials on their website [6]. To classify publications, we were guided by publication types as defined by the Medical Subject Headings thesaurus [31].

## RESULTS

We identified fifty-four studies that met our inclusion criteria, a summary of which is available in Table 1 [32–85]. Using six themes drawn and modified from FOSTER's OS Taxonomy, we identified open access, open data, and open science as the most prominent themes (Table 2).

**Table 1:** Summary of fifty-four studies identified for inclusion [32–85]

Reference	Purpose	FOSTER OS classification	Publication type	Study methods	Geographic location	Participants
(32) Anyaoku EN. Data librarianship: open data awareness, perceptions and services in medical libraries in Nigeria. IFLA Conference. 2019;1–12. Available from: http://library.ifla.org/id/eprint/2786/.	To assess open data awareness & services in Nigerian medical libraries	Open data	Journal article	Survey/questionnaire	Awka, Nigeria	Nigerian medical librarians
(33) Arning U, Blortz U, Brüggemann-Hasler B, Herrmann-Krotz G, Müller E, Zängl U. ZB MED – Informationszentrum Lebenswissenschaften: Eine wissenschaftliche Fachbibliothek versteht sich als Motor für Open Access. GMS Med-Bibl-Inf. 2019;19(1/2):1–5.	To describe open access (OA) services and programs at the German National Library of Medicine (ZB MED)	Open access	Case study	Descriptive research	Bonn, Germany	ZB MED librarians
(34) Azzam A, Bresler D, Leon A, Maggio L, Whitaker E, Heilman J, Orlowitz J, Swisher V, Rasberry L, Otoide K, Trotter F. Why medical schools should embrace Wikipedia: final-year medical student contributions to Wikipedia articles for Academic Credit at One School. Acad Med. 2017;92(2):193–200.	To assess impact of a Wikipedia course for medical students at the University of California, San Francisco (UCSF)	Open education	Journal article	Mixed methods; focus groups; interviews	San Francisco, California, United States	UCSF medical students, faculty
(35) Banks MA, Persily GL. Campus perspective on the National Institutes of Health public access policy: University of California, San Francisco, library experience. J Med Libr Assoc. 2010;98(3):256–9.	To gauge awareness of NIH's public open access policy among UCSF faculty and their views (positive and negative) about open access deposit requirements	Open access	Journal article	Survey/questionnaire	San Francisco, California, United States	UCSF faculty
(36) Bauer B. Kooperationen bei der Literatur- und Informations-versorgung von medizinischen Fakultäten und Hochschulen in Österreich. GMS Med-Bibl-Inf. 2018;18(1/2):1–10.	To describe collaboration networks in Austrian medical libraries; topics mentioned include open science, research data management, and open access	Open access; open data	Case study	Descriptive research	Vienna, Austria	Austrian universities & researchers
(37) Blobaum P. It's the law: impact and response to an unfunded open access act. Nurs Allied Health Resour Sect NAHRS Newsl. 2016;36(4):5–7.	To describe implementation of open access policy and institutional repository at Governors State University (GSU)	Open access	Case study	Descriptive research	University Park, Illinois, United States	GSU faculty; library
(38) Borghi J, Cuddy C. The Library as an active collaborator in meta-science, open science, and data science. MetaScience Symposium., 2019. Available from: https://www.metascience2019.org/poster-session/john-borghi/.	To describe Lane Medical Library's new services related to open science, data science, and scholarly communications	Open data; open science	Poster	Descriptive research	Stanford, California, United States	Stanford researchers
(39) Buys CM, Shaw PL. Data management practices across an institution: survey and report. J Librariansh Sch Commun. 2015;3(2):1–24.	To investigate how researchers at Northwestern University (NWU) currently manage data to determine their future needs regarding data management	Open data	Journal article	Survey/questionnaire	Evanston, Illinois, United States	NWU faculty & researchers
(40) Coates HL, Carlson J, Clement R, Henderson M, Johnston LR, Shorish Y. How are we measuring up? Evaluating research data services in academic libraries. J Librariansh Sch Commun. 2018;6:1–33.	To examine five case studies and have a conversation about how to evaluate and assess research data services in academic libraries	Open data	Case study	Mixed methods; descriptive research; environmental scan	Indianapolis, Indiana, United States	Five libraries, data services
(41) Deardorff A. Why do biomedical researchers learn to program? An exploratory investigation. J Med Libr Assoc. 2020;108(1):29–35.	To discuss the impact of programming workshops on computational reproducibility and researcher workflows at UCSF	Open science	Journal article	Mixed methods; survey/questionnaire; interviews	San Francisco, California, United States	Researchers at UCSF
(42) Di Salvo I, Mwoka M, Kwaga T, Rukundo PA, Ernest DS, Osaheni LA, John K, Shafik K, de Sousa AM. Open access, open education resources and open data in Uganda. Pan Afr Med J. 2015;21.	To describe open access and open data movements in Uganda and advocacy efforts of medical students	Open access; open data; open education	Report	Descriptive research	Kampala, Uganda	Ugandan medical researchers, students
(43) Federer L. Research data management in the age of big data: roles and opportunities for librarians. Inf Serv Use. 2016;36(1/2):35–43.	To discuss opportunities in big data for librarians and other information professionals to support management and preservation of research data	Open data	Case study	Descriptive research	Bethesda, Maryland, United States	NIH researchers, librarians, and informationists
(44) Féret R, Cros M. The embedded research librarian: a project partner. Liber Q J Eur Res Libr. 2019;29(1):1–20.	To describe new services developed by the Lille University Library for European and National research project coordinators	Open access	Case study	Descriptive research	Lille, France	French researchers
(45) Flitner U, Grimm S. Einführung von Open-Access-Services an der Charité – Universitätsmedizin Berlin. Ein Praxisbericht. GMS Med-Bibl-Inf. 2019;19(1/2):1–6.	To describe Germany's Charité Medical Library's support of OA funding project involving campus stakeholders	Open access	Case study	Descriptive research	Berlin, Germany	German researchers
(46) Foster ED, Coates HL. Raising the visibility of protected data: a pilot data catalog project. In Open Praxis, Open Access: Digital Scholarship in Action. Chicago, IL: ALA Editions; 2020.	To configure an existing institutional data repository as a data catalogue	Open data	Book chapter	Descriptive research	Indianapolis, Indiana, United States	Faculty, graduate students, postdocs, research staff
(47) Gordon S. Why libraries aren't dead: open access and the evolving liaison role. J Can Health Libr Assoc JCHLA. 2011;32(3):165–7.	To explore liaison roles for Canadian health sciences librarians in open access	Open access	Journal article	Descriptive research	Waterloo, Ontario, Canada	Canadian researchers
(48) Gore SA. E-science and data management resources on the web. Med Ref Serv Q. 2011;30(2):167–77.	To review websites relevant to librarians who are interested in learning more about data management	Open access; open data	Review	Descriptive research	Boston, Massachusetts, United States	UMass researchers
(49) Greyson D. Open access and health librarians in 2011. J Can Health Libr Assoc JCHLA. 2011;32(2):45–9.	To provide an overview of OA in Canada focusing on health information, updates of OA, needs of librarians, and opportunities due to OA shift	Open access	Journal article	Descriptive research	Vancouver, British Columbia, Canada	Canadian researchers
(50) Heselden M, Malliarakis KD, Lunsford B, Linton A, Sullo E, Cardenas D, LeGal M, Guzzetta CE. Establishing an open access repository for doctor of nursing practice projects. J Prof Nurs. 2019;35(6):467–72.	To develop a project repository for doctor of nursing practice students at George Washington University (GWU)	Open science	Case study	Mixed methods; environmental scan; interviews	Washington, District of Columbia, United States	Doctor of Nursing Practice (DNP) students at GWU
(51) Ignat T, Ayris P, Juan ILI, Reilly S, Dorch B, Kaarsted T, et al. Merry work: libraries and citizen science. Insights UKSG J. 2018;31:1–10.	To describe how libraries can support citizen science in the United Kingdom, Spain, European Union, and Qatar	Citizen science	Journal article	Descriptive research	Geneva, Switzerland	Citizens; librarians from UK, Spain, European Union, and Qatar
(52) Ilik V, Hebal P, Olson A, Wishnetsky S, Pastva J, Kubilius R, Shank J, Gutzman K, Chung M, Holmes K. DigitalHub: A repository focused on the future. Med Ref Serv Q. 2018;37(1):31–42.	To describe the DigitalHub project challenges and team-based approaches used to achieve project goals	Open access	Case study	Descriptive research	Chicago, Illinois, United States	Researchers at NWU
(53) Johnson LM, Butler JT, Johnston LR. Developing e-science and research services and support at the University of Minnesota Health Sciences Libraries. J Libr Adm. 2012;52(8):754–69	To describe the development and implementation of e-science and research services at Health Sciences Libraries (HSLs), Academic Health Center (AHC) at University of Minnesota (UMinn)	Open data; open science	Case study	Descriptive research	Minneapolis, Minnesota, United States	Researchers at UMinn
(54) Kafel D. Activities of regional consortia in planning e-science continuing education programs for librarians in New England. In: Special Issues in Data Management. American Chemical Society; 2012. p. 69–96. Available from: https://pubs.acs.org/doi/abs/10.1021/bk-2012-1110.ch005.	To describe e-science's impact on libraries, education programs, and research sponsored by the consortium of New England librarians	Open science	Book chapter	Mixed methods; survey/questionnaire; descriptive research	Worcester, Massachusetts, United States	New England science librarians
(55) Ketchum AM. The research life cycle and the health sciences librarian: responding to change in scholarly communication. J Med Libr Assoc. 2017;105(1):80–3.	To discuss services to support research data life cycle and scholarly communication and services related to open science	Open access; open data; open science	Journal article	Descriptive research	Pittsburgh, Pennsylvania, United States	Health sciences librarians
(56) Kipnis DG, Palmer LA, Kubilius RK. The institutional repository landscape in medical schools and academic health centers: a 2018 snapshot view and analysis. J Med Libr Assoc. 2019;107(4):488–98.	To gain a deeper understanding of the institutional repository landscape in medical schools and academic health centers	Open access	Journal article	Survey/questionnaire	Glassboro, New Jersey, United States	Association of Academic Health Sciences Libraries
(57) Kipnis DG, Palmer LA. Medical institutional repositories in a changing scholarly communication landscape. Against the Grain. 2018;30(4):33–6.	To update progress of institutional repository services provided by health sciences libraries and barriers and challenges in providing those services	Open access	Journal article	Descriptive research	Glassboro, New Jersey, United States	Researchers Association of Academic Health Sciences Libraries
(58) Lapinski PS, Osterbur D, Parker J, McCray AT. Supporting public access to research results. Coll Res Libr. 2014;75(1):20–33.	To discuss services an academic library can best provide to support the NIH Public Access Policy	Open access	Case study	Descriptive research	Boston, Massachusetts, United States	Researchers at Harvard
(59) Lawton A. Communicating the open access message: a case study from Ireland. New Review of Academic Librarianship. 2016 Jan 2;22(1):60–77.	To review OA week 2015 in Ireland through use of statistics and survey	Open access	Case study	Survey/questionnaire	Dublin, Ireland	Researchers and librarians in Ireland
(60) Lin N, Hinegardner PG. Discovering the present, preserving the past: the development of a digital archive at the University of Maryland. J Electron Resour Med Libr. 2012;9(4):247–60.	To discuss implementation of the University of Maryland Digital Archive and experiences and lessons learned across project timeline.	Open access	Case study	Descriptive research	Baltimore, Maryland, United States	Researchers and librarians at University of Maryland
(61) Lindstädt B. Management und Publikation von Forschungsdaten - Serviceleistungen einer wissenschaftlichen Bibliothek. Bibliotheksdienst. 2016;50(7):636–48.	To describe data services developed at ZB MED Library, networking with researchers, and publishing their data	Open data	Case study	Descriptive research	Bonn, Germany	Researchers at ZB MED
(62) MacMillan D. Developing data literacy competencies to enhance faculty collaborations. Liber Q J Eur Res Libr. 2015;24(3):140–60.	To describe how life sciences data was incorporated into an information literacy program to align instruction with student and faculty learning needs	Open data	Case study	Descriptive research	Calgary, Alberta, Canada	University of Calgary biology and genetics students
(63) Mani NS, Rosenzweig M, Masters CM. Improving clarity of compliance procedures associated with the National Institutes of Health (NIH) Public Access Policy (NIHPAP) via process mapping. J Libr Adm. 2015;55(2):79–91.	To describe process mapping exercise to help with NIH public access compliance at the University of Michigan	Open access	Case study	Descriptive research	Ann Arbor, Michigan, United States	University of Michigan researchers
(64) McGowan BS. OpenStreetMap mapathons support critical data and visual literacy instruction. J Med Libr Assoc. 2020;108(4):649–50.	To discuss mapathons and highlight how libraries have hosted them to support disaster relief efforts	Open data; open source	Journal article	Descriptive research	West Lafayette, Indiana, United States	Researchers, librarians
(65) Mundoma C, Ruhs N, Meth M, Glerum A, Lopez M, Julian R. Research Resource Identifiers (RRID) for core facilities and research equipment. J Biomol Tech. 2019;30(Supplement):S35.	To develop a structured citation style and tracking tool (or index) for scientific lab equipment	Open science	Poster	Descriptive research	Tallahassee, Florida, United States	Researchers, librarians at FSU, UCSD
(66) Nguyen-Truong CKY, Graves JM, Enslow E, Williams-Gilbert W. Academic and community–engaged approach to integrating open educational resources in population health course. Nurse Educ. 2019;44(6):300–3.	To describe how to use community engagement to identify open educational resources for a nursing course	Open education	Journal article	Descriptive research	Vancouver, Washington, United States	Researchers, librarians
(67) Novak Gustainis ER. Ever-evolving: introducing the Medical Heritage Library, Inc. J Med Libr Assoc. 2019;107(2):265–9.	To describe the Medical Heritage Library (MHL), a collaborative digitization and discovery organization providing open access to history of medicine resources	Open access	Journal article	Descriptive research	Cambridge, Massachusetts, United States	Harvard University Library and MHL
(68) Overgaard AK, Kaarsted T. A new trend in media and library collaboration within citizen science? The case of ‘a healthier funen.’ Liber Q. 2018;28(1):1–17.	To describe citizen science in Funen, Denmark, where the public learns about research projects and vote on grants	Citizen science	Case study	Descriptive research	Funen, Denmark	Citizens in Denmark
(69) Palmer LA. Cultivating scholarships: the role of institutional repositories in health sciences libraries. Against the Grain. 2014;26(2):24–8.	To discuss institutional repositories, project management, training, quality control, metadata management, and customer service	Open access	Journal article	Descriptive research	Worcester, Massachusetts, United States	Researchers
(70) Read KB, Surkis A, Larson C, McCrillis A, Graff A, Nicholson J, Xu J. Starting the data conversation: Informing data services at an academic health sciences library. J Med Libr Assoc. 2015;103(3):131–5.	To gather information from faculty at New York academic medical center to inform development of data services in an HSL	Open data	Journal article	Interviews	New York City, New York, United States	Researchers at NYU medical center
(71) Rethlefsen ML, Lackey MJ, Zhao S. Building capacity to encourage research reproducibility and #MakeResearchTrue. J Med Libr Assoc. 2018;106(1):113–9.	To describe one library's work to help increase awareness of reproducibility and build capacity to improve reproducibility of research	Open science	Case study	Descriptive research	Salt Lake City, Utah, United States	Researchers at University of Utah
(72) Rosenzweig M, Schnitzer AE, Song J, Martin S, Ottaviani J. National Institutes of Health public access policy and the University of Michigan Libraries' role in assisting with depositing to PubMed Central. J Med Libr Assoc. 2011;99(1):97–9.	To describe the University of Michigan libraries' roles in assisting with PMC deposition	Open access	Case study	Descriptive research	Ann Arbor, Michigan, United States	UM researchers
(73) Rusch B, Boltze J, Dierkes T, Goltz-Fellgiebel JA, Staub H. DeepGreen – DeepGold: Open-Access-Transformation – Entwicklung und Perspektiven. GMS Med-Bibl-Inf. 2019;19(1/2):1–7.	To describe Germany's DeepGreen project, an automated method for collecting article data and delivering it to repositories for OA publications	Open access	Journal article	Descriptive research	Berlin, Germany	German researchers
(74) Sayre F, Riegelman A. Replicable services for reproducible research: a model for academic libraries. Coll Res Libr. 2019;80(2):260–72.	To outline model for academic libraries' support of reproducible research based on recommendations of funders, professional societies, publishers, academic libraries	Open science	Journal article	Descriptive research	Minneapolis, Minnesota, United States	Researchers
(75) Schmidt B, Bertino A, Beucke D, Brinken H, Jahn N, Matthias L, Mimkes J, Müller K, Orth A, Bargheer M. Open science support as a portfolio of services and projects: From awareness to engagement. Publications. 2018;6(2).	To outline library's open science support as a Portfolio of Services and Projects at the University of Göttingen (UG)	Open access; open data; open science	Case study	Descriptive research	Göttingen, Germany	German researchers at UG
(76) Sheffield CL, Refolo LM, Petanceska SS, King RJ. A librarian's role in improving rigor in research—AlzPED: Alzheimer's Disease Preclinical Efficacy Database. Sci Technol Libr. 2017;36(3):296–308.	To review project of the National Institutes of Health Library (NIH Library) and National Institute on Aging in creating a database for preclinical AD research: the Alzheimer's Preclinical Efficacy Database (AlzPED)	Open access	Case study	Descriptive research	Bethesda, Maryland, United States	Researchers at NIH, NIH Library
(77) Sinn RN, Woodson SM, Cyzyk M. The Johns Hopkins Libraries open access promotion fund: an open and shut case study. Coll Res Libr News. 2017 Jan 1;78(1):32–5.	To develop a promotional open access fund to encourage authors to publish in OA journals	Open access	Case study	Descriptive research	Baltimore, Maryland, United States	Researchers at Johns Hopkins
(78) Spremberg A, Schmiel M, Hartmann K. Open Access an der Medizinischen Hochschule Hannover: Erfahrungen aus der Perspektive der Bibliothek. GMS Med-Bibl-Inf. 2019;19(1/2):1–6.	To track university medical school's open access publications and support for OA publishing	Open access	Case study	Descriptive research	Hannover, Germany	German researchers
(79) Steinrisser-Allex G, Grossmaier-Stieg K. Open Access an der Medizinischen Universität Graz – Therapieentscheidungen im Spannungsfeld zwischen Forschung und klinischem Alltag. GMS Med-Bibl-Inf. 2019;19(1/2):1–10.	To describe an open access strategy used by a medical university library in Austria	Open access	Case study	Descriptive research	Graz, Austria	Researchers at Medical University of Graz, Austria
(80) Taylor A. Libraries take on policy: support for open access and open data. Against the Grain. 2014;26(2):28–32.	To provide a review of health sciences libraries' early support for open access support and complying with the NIH Policy	Open access; open data	Journal article	Descriptive research	San Francisco, California, United States	Researchers
(81) Thomas WmJ, Blackwell L. NIH mandate one year on: how are libraries responding? Ser Libr. 2010;58(1–4):257–62.	To identify how academic libraries are responding to the NIH public access mandate and the role of institutional repositories	Open access	Journal article	Survey/questionnaire	Greenville, North Carolina, United States	Researchers
(82) Vieler A, Wöckel C. Vom Informationsversorger zum Forschungsdienstleister – Änderung der Wahrnehmung bibliothekarischer Arbeit durch Open Access an der Bibliothek Medizin/Naturwissenschaften der Universität Leipzig. GMS Med-Bibl-Inf. 2019;19(1/2):1–5.	To discuss the promotion of open access publishing at Leipzig University	Open access	Case study	Descriptive research	Leipzig, Germany	German researchers
(83) Wang H, Gainey M, Gulick AV. Carnegie Mellon's first Open Science Symposium – Themes about research data and their reuse. Poster session at the Open Science Symposium, Research Data Alliance Plenary Meeting 13. April 1–4, 2019; Philadelphia, PA. Available from: https://kilthub.cmu.edu/articles/poster/Carnegie_Mellon_s_first_Open_Science_Symposium_-_Themes_about_research_data_and_their_reuse/7928714.	To describe development of an Open Science symposium to raise awareness and support for open research practices and data sharing at Carnegie Mellon University (CMU)	Open data; citizen science; open science; open source	Poster	Descriptive research	Pittsburgh, Pennsylvania, United States	CMU faculty, grad students, postdocs in neuroscience, biology, computer science, and engineering
(84) Wright RA. Developing a suite of online learning modules on the components of next-generation sequencing projects. Med Ref Serv Q. 2020;39(1):90–9.	To describe project funded by the NIH Big Data to Knowledge initiative to develop open education modules at Johns Hopkins	Open data	Journal article	Descriptive research	Baltimore, Maryland, United States	Researchers, informationists Johns Hopkins School of Medicine
(85) Milewska A, Wiśniewska N. Poland's first open library research data–from theory to practice. Med Libr Forum. 2019;12(1):2–8.	To describe the implementation of the Polish Medical Platform portal to support OA and advocacy of open science	Open access; open data; open science	Journal article	Survey/questionnaire	Gdańsk, Poland	Polish researchers

**Table 2:** Fifty-four studies categorized using FOSTER OS Taxonomy

FOSTER Open Science Taxonomy themes	Number of studies (%)
Open access	29 (54%)
Open data	23 (43%)
Open science	13 (24%)
Open education	3 (6%)
Citizen science	3 (6%)
Open source	3 (6%)

***Note:*** Studies were assigned between one and four OS labels, with most receiving one

**Figure 2:**
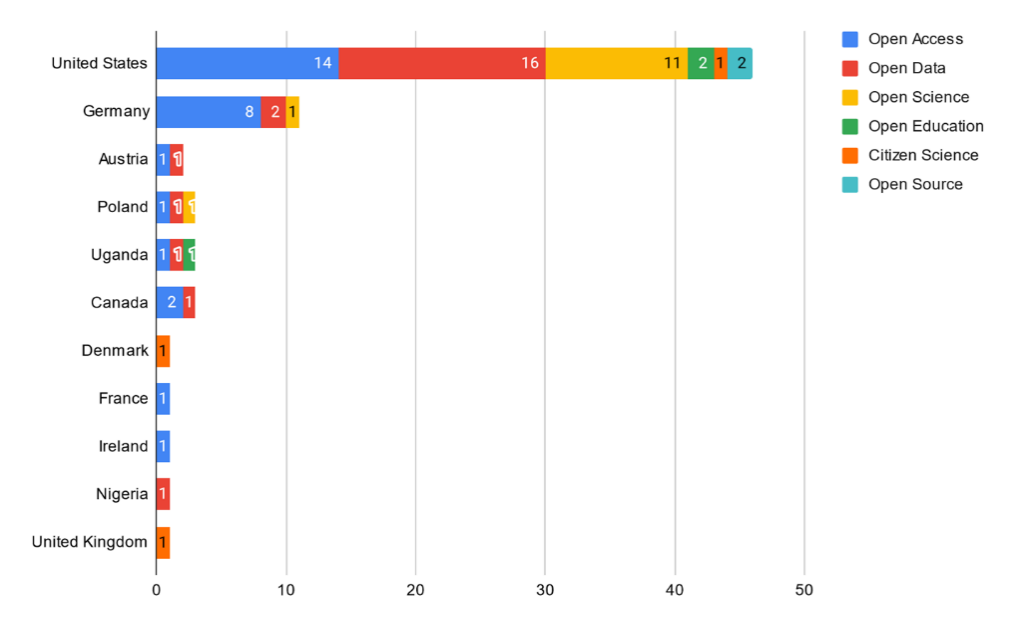
HSL-specific OS publications by country

Most studies were published in English (n=46, 85%), and the rest were in German (n=8, 15%). The studies originated in or had corresponding authors from North America (n=38, 70%), Europe (n=14, 26%), and continental Africa (n=2, 4%) (Figure 2).

**Figure 3:**
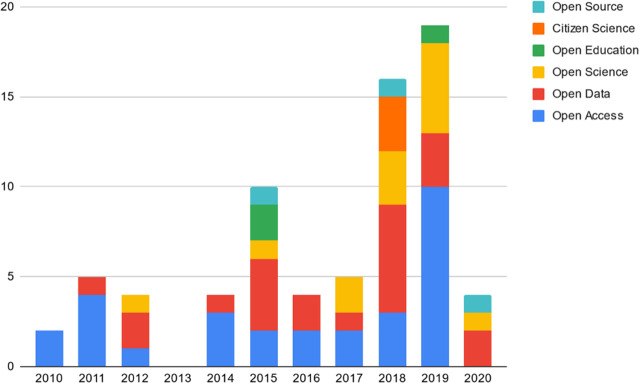
HSL-specific OS publications by year

A majority of studies (n=41, 76%) were published from 2016 to 2020, with a high frequency (n=28, 52%) being published from 2018 to 2020 (Figure 3).

Most of our studies fit the definition of a case study, “a descriptive and exploratory analysis of a particular library, service or event.” We adopted the definition of research articles as “the predominant publication type for articles and other items indexed for NLM databases” [4, 31] unless one of the other categories was more appropriate.

Publication types included case studies (n=25, 46%), research articles (n=21, 39%), conference posters and abstracts (n=5, 9%), book chapters (n=2, 4%), and reports (n=1, 2%). We classified publications by study method including descriptive or narrative-based approaches such as perspective pieces, commentaries, and editorials (n=41, 76%); surveys, questionnaires, and interviews (n=8, 15%); and mixed methods (n=5, 9%).

Studies were published in 27 interdisciplinary scholarly and journal sources. Several studies appeared in the *Journal of the Medical Library Association* (n=8, 15%) and the *GMS Medizin-Bibliothek-Information* (n=7, 13%). Bibliographic databases containing the most studies were LISTA (n=22, 41%), Web of Science (n=12, 22%), CINAHL (n=8, 15%), and MEDLINE (n=7, 13%). We include a list of 54 studies and journal sources, traced back to their original database, in Supplemental file S5.

**Figure 4:**
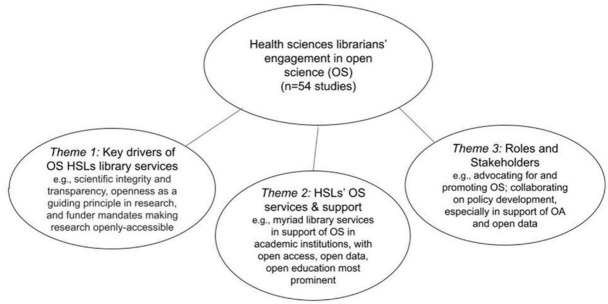
Key OS themes

Our thematic analysis reveals the multidimensional nature of HSLs' support of open science. We clustered findings into three themes based on our research questions: 1) key actions, barriers, and drivers of OS in HSLs; 2) types of OS services described; and 3) roles and stakeholders involved in providing HSL-specific support in OS (Figure 4). The relevant subthemes are clustered below each main theme, and the number of studies addressing each subtheme is indicated in brackets.

### Theme 1: Key actions, barriers, and drivers of OS HSL library services

We identified the following key actions and drivers for HSLs' engagement in open science: aligning OS with the core principles and values of HSLs (n=23, 43%); participating in national initiatives to improve openness such as Europe's Plan S Initiative for Open Access Publishing [86] and the US Office of Science and Technology Policy's memorandum on increasing access to federally funded research (n=16, 30%) [33]; helping researchers to comply with funder mandates such as the NIH Public Access Policy, EU Open Science Policy, and NIH Rigor and Reproducibility requirements (n=11, 20%); undertaking OS initiatives at the campus level, for example designing library services to align with institutional drivers such as university policies, working with faculty members and instructors to teach OS and its principles, and developing OS-specific infrastructure (n=8, 15%) or cross-institutional projects to initiate or improve OA (n=4, 7%); and initiating OS services because other libraries were doing so (n=3, 6%). Specific barriers to providing OS services were not explicitly mentioned.

### Theme 2: HSLs' OS services and support

#### Advocacy and outreach for OS services

HSLs engage in advocacy and outreach for OS in many ways, with most examples coming from academic institutions, including advocating for OA and OS in the academic health context, including supporting and promoting OA funds (n=7, 13%); showcasing OA and OS research efforts on campus by making presentations, holding workshops, and pursuing cross-institutional initiatives (n=4, 7%); coordinating various stakeholders on campus to support the adoption of open publishing models as well as open educational resources (n=4, 7%); serving as mediators and experts in local open data efforts in academic institutions or being members in national organizations (n=2, 4%); and promoting the principles of citizen science by building connections between researchers and local communities, scheduling citizen outreach, and codeveloping community-researcher-based networks (n=2, 4%). We found no evidence of advocacy or outreach for open source–related services.

#### Open access services and resources

HSLs help to promote OA to researcher communities by offering the following services and resources: raising awareness of OA principles and their adoption (n=8, 18%); developing infrastructure to assist in depositing OA publications for policy compliance (n=5, 9%); managing institutional OA publications (n=2, 4%); developing institutional repositories (n=5, 9%) and digital archives (n=2, 4%); creating and maintaining library guides to disseminate key library services for OA (n=5, 9%); and encouraging compliance with national funder policies, specifically the NIH Public Access policy and EU Open Access policy (n=4, 7%).

#### Open data services and resources

HSLs help to promote open data by offering the following services and resources: providing support for the reuse and sharing of open data (n=6, 11%); offering digital object identifier (DOI) services for datasets (n=2, 4%); and providing technical and advisory support for the Open Science Framework (n=2, 4%).

HSLs provide instructional and training sessions on the following open data topics: finding open data (n=5, 9%); managing and using open data (n=4, 7%); sharing data (n=4, 7%); introductions to open data (n=3, 6%); and, FAIR training (n=1, 2%) [87].

HSLs have developed the following resources to support open data: repositories to store and share institutional research data (n=6, 11%); data catalogs to improve the discovery of institutional research data that cannot be publicly shared (n=2, 4%); and open data LibGuides (n=2, 4%).

#### Open science resources and services

HSLs help to promote the broader theme of OS by offering the following services and resources: coordinating and organizing OS conferences, meetups, and events (n=3, 6%); and supporting campus OS policies (n=2, 4%).

In addition, HSLs provide OS instructional and training services on the following topics: research reproducibility, transparency, and OS best practices and principles (n=4, 7%); data science training, specifically the use of R and Python (n=2, 4%); OS-specific tools, specifically research protocol sharing software (n=1, 2%); and adoption, use, and attribution of standards used in research projects (n=1, 2%).

HSLs provide OS support on institutional and grant-funded research projects including: collaborating on initiatives to improve research workflow infrastructure and guide development and sharing of research outputs (n=3, 6%); and developing infrastructure to support data citation and the implementation of data standards used for research projects (n=3, 6%).

#### Citizen science services and resources

HSLs help to promote citizen science by offering the following services and resources: providing access to tools and technology to help community members better understand science and ongoing research projects at the librarian's institution (n=1, 2%); developing a citizen science toolkit for researchers and community members (n=1, 2%); training citizens on institutionally developed research projects where citizens then vote on which projects receive grant funding (n=1, 2%); and teaching the value of citizen science (n=2, 4%), principles of information literacy (n=1, 2%), and new methods of data collection (n=1, 2%).

#### Open education services and resources

HSLs help to promote open education by offering the following services: holding consultations with faculty and students to select quality open education resources for their own teaching responsibilities (n=2, 4%); supporting medical students making contributions to and editing medical articles in Wikipedia (n=1, 2%); utilizing open education resources to provide to medical students (n=1, 2%); and providing training on copyright guidance and fair use (n=1, 2%). We found no evidence of resources or tools developed to support open education.

#### Open source services and resources

HSLs provide the following open source services and support: hosting open source mapathons (n=1, 2%); offering training on the availability and use of open source data and software (n=1, 2%); and using Wikipedia as an open source wiki platform for collaboration and source of evidence-based medical information (n=1, 2%). We found no evidence across the included studies that the OS services and resources described above were assessed or evaluated.

### Theme 3: Roles and stakeholders involved in providing HSL OS services and support

In addition to their teaching and consultation roles in OS, HSLs assume several key and prominent roles within their institutions by offering the following services and support: advocating for and compliance of OS throughout the research process (n=20, 37%); collaborating on institutional OS-related policy development, specifically policy as it relates to OA and open data (n=6, 11%); building community within institutions with respect to OS principles and, in Europe, establishing connections with ongoing OS national initiatives (n=4, 7%); and providing metadata support (n=1, 2%) and digital preservation (n=1, 2%) for OA and open data initiatives in academic institutions.

While the types of roles articulated across the included studies were described broadly, there was no mention of specific job titles or formal institutional responsibilities that were established to carry out these roles.

HSLs collaborate and establish partnerships with several stakeholders to provide OS services and resources, including: university leadership (n=11, 20%); research support units in academic institutions (n=9, 17%); national partners, specifically federal and local governments participating in OS initiatives (n=6, 11%); information technology (IT) departments and personnel (n=7, 13%); CTSA (Clinical and Translational Science Award) teams or hubs (n=5, 9%); faculty in biomedical and health programs (n=5, 9%); and other campus and local libraries (n=4, 7%).

## DISCUSSION

This scoping review identified fifty-four studies with authors from eleven countries and three continents who worked mostly as government and academic HSLs. The published literature has increased considerably since 2010, with 52% of articles published since 2018, signaling the topic's increased importance for HSLs. We extracted a total of fifty-seven unique sub-themes, highlighting a range of OS services provided by HSLs. Based on our analysis, we identified the following areas that could benefit from further exploration: 1) geographic concentration of OS support by country and continent, 2) social justice issues related to OS, 3) OS-specific gaps to address in future research, and 4) the lack of evidence to support the success of HSL OS services.

### Geographic concentration of OS support

Our review highlights trends and variations in how HSLs from different countries implement OS services. Studies published in European countries, for example, revealed strong coordination between national efforts, such as the European OA policy and release of Plan S for OA publishing, and adoption of these efforts at the institutional level. Furthermore, HSLs coordinated activities such as working with their governments to identify and implement OS services; contributing to policy development at an institutional level and informed by national policy; and, in collaboration with national partners, developing infrastructure (e.g., institutional repositories) and resources (e.g., OA reporting for policy compliance). Two studies from continental Africa (Nigeria and Uganda) demonstrated strong alignment with national initiatives. The Uganda study in particular outlined a project that brought together students and researchers at a government-sponsored event to explore open education resources.

By comparison, North American studies predominantly published by US institutions demonstrated that HSLs use national policies (e.g., NIH public access policy) to justify their development of OS services, but these services are not typically created in direct coordination with national governments and stakeholders. North American HSLs may find it useful to look at examples in Europe to determine how OS services could be developed alongside national partners. Further, given the shortage of perspectives from regions in Oceania, Latin America, and continental Africa, HSLs from these jurisdictions should be encouraged to publish their research more generally and also to highlight best practices that are emerging in creating those important partnerships.

### Open science and social justice

Only one of the fifty-four studies mentioned OS's potential to advance equity and social justice perspectives [88]. Given that one of the goals of OS is to make research results more widely available, we were surprised to learn that equity and social justice were not mentioned more broadly as drivers of OS services. Many biomedical researchers and clinicians are increasingly invested in anti-racist work, so there is an opportunity to highlight OS practices as an alternative to the traditionally closed, white supremacist models of scholarly publishing [89]; however, there is some concern that OS also suffers from similar exclusive practices [90]. HSLs with institutional diversity, equity, and inclusion (DEI) initiatives might consider aligning OS services in support of those programs by advocating for DEI funds to support OA publishing fees for early career and underrepresented researchers. Given the evidence that they are more likely to be excluded in OS, there should be greater focus on supporting women, minority groups, and Black, Indigenous, and People of Color (BIPOC) [89]. Another important consideration is to make more explicit the connections between OS and equity in DEI programming by providing opportunities to groups historically marginalized or excluded from open research due to disparities in power and privilege [91].

### OS-specific gaps in the literature

Our review highlights numerous gaps in the HSL OS-specific literature. First, while there was a high frequency of HSL OS studies in academic settings, we found no studies in clinical or hospital settings. While this may reflect the often smaller budgets and limited staff time at these institutions, we argue that OS support is especially important for under-resourced institutions, where researchers and clinicians often publish in journals they cannot afford to license and would benefit from more awareness of OA models. We recommend that academic HSLs partner with hospital librarians in their area to collaborate on OS services to support mutual goals. We suggest that citizen science and OA might be good places to start.

Second, only a small number of studies discussed providing targeted OS support to specific groups (e.g., nursing, basic science), while the remainder focused on broad, institution-wide services. We recommend that future research focus on collaborating, incorporating, aligning, and evaluating HSL OS services with user communities that have a logical connection to OS, such as population and public health.

The third gap highlights the need for additional research into HSL-supported open education and open source services. The role of open education resources in health sciences degree programs, and how they can be deployed to support students and faculty, should be more fully explored. As open education services continue to show benefits in providing equitable access to information resources, future HSL literature should examine the development and implementation of these services. Similarly, empirical studies are needed about how, where, when, and with whom open source tools and resources are provided by HSLs throughout the research lifecycle, as evidence to support these services was largely absent.

Finally, our review highlights a lack of publications in citizen science and its potential to further patient-centered research [92]. Despite the two examples we found, there were few examples of citizen-specific or community-based partnerships. As HSLs continue to provide library services and promote OS principles, we believe that more concerted efforts are needed to engage local communities and individuals (e.g., patients, research participants, neighborhood organizations) in projects at an institutional level. A shift toward more support for citizen science would also demonstrate HSLs' commitment to advocating for OS beyond their institutions and help to connect researchers with community partners and patient advocacy groups to ensure research results are shared to maximize equity and impact.

### Lack of evidence of the success of HSL OS services

Our review highlights the limitations in the HSL literature related to evidence-based OS practices. While the included studies describe how HSLs are providing OS services, more empirical research is needed to demonstrate the impact of these services and the costs versus benefits of these services over time, including rigorous assessment and sustainability of library service models. Due to a lack of robust study designs, we could not assess OS services provided by HSLs or determine for whom the OS services were intended beyond general mention of students and faculty. Interestingly, many studies stated in their recommendations the need for additional support from their institutions to advance OS and its principles. Empirical evidence gathered from HSL OS services could help influence institutional support for additional HSL resources and personnel.

### Limitations

This scoping review has some limitations. By limiting our searches to a finite set of bibliographic databases and websites, we acknowledge that we may have excluded important perspectives of HSL OS services, particularly from continental Africa and South America. In addition, by limiting the date range of our searches from 2010 to 2020, we may have missed studies published before 2010. We opted to limit our searching to the previous ten years to ensure that the body of literature would address OS services more broadly, rather than emphasize open access, which was the primary OS service described before 2010. To account for any relevant OA studies published before 2010, we quote from and cite those papers in our introduction.

### Conclusion

HSLs are engaged in providing various OS library services and programs and play prominent roles in advancing OS practices and principles worldwide. However, more formal studies should be conducted to assess their support of OS researchers and to determine the best and most sustainable library services and programs going forward. Future research should focus on researchers' needs in OS and robust assessment of library service models specifically designed to meet them. Furthermore, HSLs can amplify OS by adopting and promoting more rigorous and transparent research practices of their own. Future research should also examine HSLs' engagement in OS through equity and social justice perspectives.

## SUPPLEMENTAL FILES

**S1.** Grey literature

**S2.** Search concept blocks

**S3.** Medline search strategy (5 March 2020)

**S4.** Elements captured in the data extraction form

**S5.** Summary of journals represented in fifty-four included papers
