# Supplementary material for: Health sciences librarians' engagement in open science: a scoping review
Source: J Med Libr Assoc. 2021 Oct 1;109(4):540–60. doi: 10.5195/jmla.2021.1256 (PMC8608193; doi:10.5195/jmla.2021.1256)
Supplement: Supplementary file 1 — S2. Search concept blocks [file jmla-109-4-540-s02.docx]

## **S2. Search concept blocks**

Keywords, synonyms and associated controlled terms

| **Concept #1**  *Health sciences librarians*  *__________________________*  “health sciences librar*”  “health librar*”  “health information professional or health information specialist”  “hospital librar*  “informationist”  “medical librar*” | **Concept #2**  *Open science*  *__________________________*  “open science”  “open research”  “open scholarship”  “open access”  “open data”  “data publishing”  “data sharing”  “sharing data”  “pre-registration”  (research AND transparen*)  reproducib*  replicab*  “open educat*”  “open licensing”  “open metrics”  “open notebook*”  “open pedagog*”  “open peer review”  “open practice*”  “open protocol*”  “open source”  “open textbook*”  “lab notebook*”  ((code OR data OR software) AND availab*)  (software OR data) AND carpentr*  ((computation* OR programm*) AND (R OR Python OR Jupyter OR markdown)  Xenodo OR Open Science Framework, OR Github, etc. | **Concept #3**  *Actions, roles, support, outcomes ____________________*  activit*  advis*  advoca*  benefit*  collabor*  competenc*  consult*  cost*  course*  creat*  engag*  expert*  guid*  initiat*  instruct*  knowledge  lead*  member*  opportunit*  participant*  project*  provid*  recommend*  role*  search*  skill*  specialist* |
| --- | --- | --- |
