# Supplementary material for: Health sciences librarians' engagement in open science: a scoping review
Source: J Med Libr Assoc. 2021 Oct 1;109(4):540–60. doi: 10.5195/jmla.2021.1256 (PMC8608193; doi:10.5195/jmla.2021.1256)
Supplement: Supplementary file 2 — S3. Medline search strategy (5 March 2020) [file jmla-109-4-540-s03.docx]

## **S3. Medline Search Strategy (5 March 2020)**

Database: Ovid MEDLINE(R) and Epub Ahead of Print, In-Process & Other Non-Indexed Citations, Daily and Versions(R) <1946 to March 5, 2020>

1. exp access to information/ (7707)
2. open access publishing/ (440)
3. "open science".mp. (1118)
4. "open research".mp. (474)
5. "open scholarship".mp. (4)
6. "open access".mp. (7815)
7. "open data".mp. (1594)
8. "open licens*".mp. (35)
9. "open metric*".mp. (0)
10. "open knowledge".mp. (17)
11. "citizen science".mp. (1297)
12. "open publishing".mp. (6)
13. "data publishing".mp. (59)
14. "data access".mp. (1192)
15. "data curation".mp. (913)
16. "data intensive".mp. (512)
17. "data plan*".mp. (192)
18. (data adj3 re*us*).mp. (2226)
19. "data sharing".mp. (4252)
20. "data visual*".mp. (2421)
21. "sharing data".mp. (760)
22. ("meta science" or metascience).mp. (67)
23. (pre-registration or preregistration).mp. (2322)
24. (research and transparen*).mp. (24189)
25. repeatability.mp. (26723)
26. reproducib*.mp. (527382)
27. replicab*.mp. (4429)
28. replicat*.mp. (342922)
29. open education.mp. (27)
30. "open lab*".mp. (46405)
31. "open pedagog*".mp. (1)
32. open notebook*.mp. (5)
33. open peer review.mp. (100)
34. "open protocol*".mp. (50)
35. open source.mp. (12320)
36. open textbook*.mp. (5)
37. lab notebook*.mp. (51)
38. "research notebook*".mp. (4)
39. ((code or data or software) and (availab* or shar*)).mp. (628841)
40. ((software or data) and carpentr*).mp. (52)
41. ((computation* or programm*) and (R or Python or Jupyter or markdown)).mp. (16762)
42. (Zenodo or Github or "open science framework").mp. (1597)
43. or/1-42 (1575608)
44. (academic adj5 health librar*).mp. (4)
45. (health adj3 information specialist*).mp. (40)
46. (health adj3 information professional*).mp. (113)
47. (health science* adj5 librar*).mp. (1341)
48. health librar*.mp. (857)
49. informationist*.mp. (82)
50. medical librar*.mp. (2759)
51. science librar*.mp. (230)
52. hospital librar*.mp. (606)
53. exp libraries/ (10126)
54. librarians/ (1060)
55. or/44-52 (5378)
56. 43 and 55 (540)
57. limit 56 to yr="2010 -Current" (302)
