# Supplementary material for: Health sciences librarians' engagement in open science: a scoping review
Source: J Med Libr Assoc. 2021 Oct 1;109(4):540–60. doi: 10.5195/jmla.2021.1256 (PMC8608193; doi:10.5195/jmla.2021.1256)
Supplement: Supplementary file 3 — S4. Elements captured in the data extraction form [file jmla-109-4-540-s04.docx]

## **S4. Elements captured in the data extraction form**

We used the following elements along two main groupings to extract data from papers:

1. Screening form article elements

- A full citation (with author, title, journal, year) in Vancouver style
- Countries of authors
- Publication type (peer-reviewed; published/unpublished report, etc.)
- Publication source (journal/website)

1. Study details

- Study design/methodology/type
- Aim(s)/objective(s) of study
- Study locations/countries/settings
- Population(s) studied
- Key findings
  - Description of open science program/service implemented
  - How library service was initiated
  - Evaluation methodology used
  - Impact of program/service
  - Institutional integration described

Recommendations, if any, made by authors
