# Supplementary material for: Health sciences librarians' engagement in open science: a scoping review
Source: J Med Libr Assoc. 2021 Oct 1;109(4):540–60. doi: 10.5195/jmla.2021.1256 (PMC8608193; doi:10.5195/jmla.2021.1256)
Supplement: Supplementary file 4 — S5. Summary of journals represented in fifty-four included papers [file jmla-109-4-540-s05.docx]

## **S5. Summary of journals represented in fifty-four included papers**

| **Journal title** | **# of papers** | **Indexing** |
| --- | --- | --- |
| Journal of the Medical Library Association | 8 | CINAHL, MEDLINE, Web of Science |
| GMS Medizin-Bibliothek-Information | 7 | LISTA |
| Against the Grain | 3 | LISTA |
| LIBER Quarterly | 3 | LISTA |
| Medical Reference Services Quarterly | 3 | CINAHL, MEDLINE |
| College & Research Libraries | 2 | LISTA, Web of Science |
| Journal of the Canadian Health Libraries Association | 2 | CINAHL |
| Journal of Librarianship & Scholarly Communication | 2 | LISTA |
| Academic Medicine | 1 | MEDLINE, Web of Science |
| Bibliotheksdienst | 1 | LISTA |
| College & Research Libraries News | 1 | LISTA |
| Conference abstracts:   - Carnegie Mellon Open Science Symposium - IFLA Conference - Stanford MetaScience Symposium | 1  1  1 | Google, Google Scholar |
| Chapter in book (Foster and Coates) | 1 | Google Books |
| Health Information and Libraries Journal | 1 | CINAHL, MEDLINE, Web of Science |
| Information Services and Use | 1 | LISTA |
| Insights the UKSG journal | 1 | Web of Science |
| Journal of Biomolecular Techniques | 1 | Embase, MEDLINE |
| Journal of Electronic Resources in Medical Libraries | 1 | CINAHL, LISTA |
| Journal of Library Administration | 1 | LISTA, Web of Science |
| Journal of Professional Nursing | 1 | CINAHL |
| NAHRS Newsletter | 1 | Google |
| Medical Library Forum | 1 | Google |
| New Review of Academic Librarianship | 1 | LISTA |
| Nurse Educator | 1 | CINAHL, MEDLINE. Web of Science |
| Pan African Medical Journal | 1 | Embase, Web of Science |
| PLoS One | 1 | MEDLINE, Web of Science |
| Publications | 1 | Web of Science |
| Science & Technology Libraries | 1 | LISTA, Web of Science |
| Serials Librarian | 1 | CINAHL, LISTA, Web of Science |
| Special Issues in Data Management, American Chemical Society (book chapter) | 1 | Google Scholar |
